# Hospital Meetings

**Published:** 1904-03-19

**Authors:** 


### HOSPITAL MEETINGS.

### CITY OF LONDON HOSPITAL FOR DISEASES OF THE CHEST.

The fifty-sixth annual meeting of this hospital was held on Thursday, 10th inst., at Gresham College. The chair was taken by Sir Edward Sassoon, M.P., treasurer.

In submitting the report and accounts for 1903, the chairman said he regretted that the attendance of Governors was a somewhat small one, but he concluded that it argued satisfaction with the conduct of affairs, and need not, therefore, be considered as a discouraging sign. He thought it would be generally agreed that the record for the year was, on the whole, one of steady and continuous progress. The erection of the Nurses' Home, a matter which, the committee felt, would brook no further delay, had been begun. The plans and specifications, with estimates, were so prepared that additions to the portion under construction, providing for not less than six additional bedrooms, could be made in sections at a cost of between £400 and £500 for each section. The section now being built would contain bedroom accommodation for 31 nurses, and the heating apparatus, lavatories and baths for the whole building, which, when complete, was intended for 40 nurses. The sum required for completing and equipping the whole was between £5,000 and £7,000. Until these rooms were built, it was impossible to add to the nursing staff, and with the present nursing strength it was equally impossible to bring the hospital up to the required number of beds, viz., 164. At present, 24 beds were closed for want of funds. This being the position of affairs, he thought the Governors would agree that the committee had met the necessities of the case in the best manner open to them. The receipts for the year amounted to £10,274, and it was exceedingly gratifying to observe that annual subscriptions had increased by the substantial sum of £110; donations, however, showed a slight decrease of £17; the Hospital Sunday Fund had increased their donation by £147, and King Edward's Hospital Fund had again given £1,500. In addition, he was glad to announce that this Fund had given a special donation of £500. Legacies, which in 1902 amounted to £937, fell during the year under review to £552. This was a source of anxiety to the committee, for while the ordinary and more or less reliable sources of income for each year amounted to about £6,000, the total expenditure reached the sum of £12,000, and in order to make both ends meet, the committee were reluctantly obliged to borrow from the bankers the sum of £1,500. With regard to the donation just referred to from King Edward's Hospital Fund, the committee confessed that they would have liked it to have reached a higher figure, and he was not without hope and belief that those who were charged with the administration of that Fund might prove to be so well satisfied with the general condition of the hospital, and the way in which the improvemnets were being carried out, that they might, next year, see their way to increase their donation. He could not too strongly impress upon the supporters of the hospital the importance that should be attached to the generous awards from this and the Sunday and Saturday Funds, for not only were they contributed to by every class of the community, but it must also be very gratifying to the subscribers to these funds to know that the awards were only given after most careful and searching inquiry. This implied the complete satisfaction of the dispensers of these funds with the work, management, and administration of this poor but deserving East End hospital. In conclusion, he wished, as chairman of that meeting, to express the indebtedness of the hospital to the medical staff, and to their hon. solicitor, Mr. Mellor, through whose instrumentality a legacy of some £3,000 was shortly to be handed over to the hospital.

Mr. F. C. Capel having seconded the motion, the report was unanimously adopted.

Sir Edward Sassoon having been re-elected treasurer, Mr. Stratford, representing the Albert and Victoria Friendly Society, suggested that possibly a larger number of working men supporters of the hospital might be able to attend the annual meetings if a preliminary notice were sent to their secretary, in addition to the insertion of the advertisement in the principal daily papers, and the chairman in reply promised that the matter should receive the consideration of the committee; personally he saw no difficulty about such a notice being sent.

The meeting closed with the usual votes of thanks, including special mention of the ladies' committee, the authorities of Gresham College for the use of the hall, and of the chairman, Sir George Wyatt Truscott, through whose efforts £2,000 was raised at the Festival Dinner in May last.

### HAMPSTEAD GENERAL HOSPITAL.

The twenty-second annual general meeting of members of the Institute was held at the Town Hall, Haverstock Hill, on Saturday, 12th inst., when in the absence, through illness, of Mr. Malcolm, the chair was taken by the Mayor, Dr. E. Collingwood Andrews. There was a very fair attendance, including a number of ladies.

In proposing the adoption of the report and financial statement, the chairman said the progress and continued usefulness of the hospital must be evident to all who read the report for 1903. It was worthy of remark that although the size of the building remained the same as before, by a slight re-arrangement of space it had been possible to increase the accommodation by two beds. There had been 387 in-patients and 2,460 out-patients during the year. A striking evidence of the usefulness of the institution was shown by the fact that the handsome donation of 100 guineas had been made by the contractors of the Tube Railway, whose workpeople were among those who received the benefits of hospital treatment. The total receipts were £2,536, including grants from the Sunday and Saturday Funds of £227 10s. and £91 4s. respectively, and a special donation of £226 2s. from the Hampstead Coronation Commemoration Fund, in addition to £1,500 apportioned to the Building Fund. Although no grant towards general maintenance was received from King Edward's Hospital Fund, a further sum of £1,000 was awarded to the Building Fund. The sum of £6,975 had now been received towards that fund, and the hospital was approaching a very important chapter in its history. It was possible that before another annual meeting the new building might be completed. After very great difficulties a site had been selected, which if it was not an ideal one, was one which, in his opinion, woud prove most efficient for the purpose. The greatest care and forethought had been exercised in its selection, and it was now the duty of the subscribers and supporters to stand by the council and aid them in every possible way to carry out the work before them. It was very encouraging to know that the Lord Mayor of London had promised to take the chair on the occasion of the biennial dinner, and the very least that could be done to show their appreciation of his kindness was to attend that dinner and support him by making up as long a list of subscriptions as possible. He himself intended to do this, and to hand it to the chairman to add to his list. He was glad to be in a position to announce that the contract for the first portion of the new building, providing 50 beds and the administrative offices, had been let, and the agreement having been signed, the work was in progress. There remained, however, a sum of £14,000 including the cost of the out-patients' department, estimated at £2,000, still to be raised; and in order that the completion might not be delayed, it would be necessary during the present year to make a strenuous effort. Generous help had been received from outside the borough, and he earnestly appealed to the residents to do their share. Festival dinners were very useful things in their place, but he thought they ought not to be necessary for the maintenance of a charitable institution the claims of which everyone ought to recognise. He did not like spasmodic efforts, and he had determined to set on foot an inquiry into the financial needs of the charitable institutions of the Borough, and to find out whether it was possible to put them on a satisfactory basis. Such a basis could only be provided by an adequate subscription-list. He felt that, as mayor, it was laid upon him to try and awaken the residents to the needs of the local institutions. It would probably be found that the same names occurred in nearly every subscription-list, and that a wider basis must be worked upon. Those who never contributed to any of the local charitable institutions must be appealed to, and he had in his mind such a centralised method as that started in Liverpool recently. At any rate, even if that were not found possible or desirable in Hampstead, he hoped the time was not far distant when the hospital would be established on a sound financial basis.

Mr. Collins, in seconding the motion, drew attention to the urgent need for a new out-patient department, the present one being most inadequate to cope with the local needs. The motion was carried.

Alderman Hanhart said the support received from the residents was very small compared with the high rateable value of the Borough, and his remarks were endorsed by Colonel Grant Gordon, who expressed himself as having been struck with the contrast between that locality and Windsor, where, he alleged, everyone contributed to the hospital.

The report of the Ladies' Association was read by Mr. Blyth, who stated that the members had sub-divided the neighbourhood and canvassed from house to house, their efforts resulting in £400, in addition to obtaining donations for the Building Fund. More members were urgently required in order that the work might be extended in scope.

The medical report having been submitted, the meeting closed with the usual votes of thanks, special mention being made of the matron, Mrs. Ebbetts, who has retired after 17 years' valuable service. The post has been filled by Miss Gregory, who was ward sister for eight years.

### ROYAL DENTAL HOSPITAL.

The annual general meeting of the Governors of the Royal Dental Hospital of London was held on Thursday, March 10th, the chair being occupied by Mr. Richard Winch, a trustee and vice-president. The attendance was not large. The report and accounts for 1903 having been unanimously adopted, the Chairman commented on the enormous number of cases dealt with—viz., 97,830, the number being 8,495 in excess of those for the previous year. Of these the anæsthetic cases were 35,619, an increase of 3,998. The increase of work implied increased working expenses, and it had been found necessary to appoint two paid house anæsthetists in addition to the honorary staff, in order to deal adequately with the numbers applying. He asked that subscribers should encourage patients to attend in the mornings, in order to avoid disappointment and the possible limiting of admissions. The total sum received for the general fund during the year was £3,409, against £3,819 in the previous year. There was a very gratifying increase of £109 in annual subscriptions, while general donations, apart from those of life governors, showed an increase of £20. The Hospital Sunday and Saturday funds contributed £195 and £120 7s. respectively, the amount received from King Edward's Hospital Fund being £300, as a special contribution towards the liquidation of the debt on the building fund. A legacy of £300 from the executors of the late Mr. Richard Bishop had been similarly applied, and he earnestly appealed to the subscribers and the public to enable the committee to pay the yearly sum of £3,000 for interest and part repayment of the loan for building the new hospital. In answer to the appeal made last year for funds to pay for the new operating-chairs, £962 2s. 6d. had been received, leaving £414 13s. still owing. Donors of chairs became life-governors of the hospital, and in addition to the usual privileges, were entitled to have their names affixed to the chairs presented. The saving of pain to those suffering from dental troubles must be evident to all, and when it was remembered that a very large number of persons, including many children, were by means of this institution thoroughly equipped for the battle of life, he thought the work must commend itself to the charitably disposed. The debt of £55,000, spread over 30 years, was a source of great anxiety to the committee, and he hoped that the facts to which he had drawn attention would result in the lightening of their burden.

A resolution of condolence with the relatives of the late Mr. J. Smith Turner, consulting dental surgeon and a vice-president of the hospital, was passed. Other business having been transacted, the meeting closed with a vote of thanks to the chairman.

### THE FRENCH HOSPITAL AND DISPENSARY.

The annual meeting of the French Hospital was held on Thursday, the 10th inst. In the absence through illness of the President, Dr. Vintras, the chair was taken by Mr. Ernest Rüffer, who remarked that this was the first annual meeting from which the president had been absent, during a period of something like 30 years, and the meeting accordingly resolved that a vote of sympathy with Dr. Vintras in his illness be accorded. The report, which was unanimously adopted, stated that 844 in-patients and 5,211 out-patients had been attended. The average number of days of residence was 20,234. There had been 12,923 consultations. In spite of incessant appeals to the public on all sides the receipts attained a total of £6,704, being an increase of £1,317 on the previous year. This total included a legacy of £800 under the will of Mons. Auguste Hachlin, and portions of the Auchois legacy amounting to £1,590. Ordinary expenses were £4,238, while the extraordinary expenses, including repairs inside the hospital, improvements and refitting of the operating theatre and mortuary, a new boiler, and sundry expenses connected with the greatly appreciated visit of M. Loubet, etc., amounted to £360. The ordinary income was £4,312, of which £845 was made up of subscriptions and £2,812 of donations.

Among the latter were £80 from M. Loubet, £100 as a special donation from King Edward's Hospital Fund, and £346 13s. 4d., and £105 10s. from the Sunday and Saturday Funds respectively.

The usual votes of thanks having been passed, the meeting came to an end.

